# E11/Podoplanin Protein Stabilization Through Inhibition of the Proteasome Promotes Osteocyte Differentiation in Murine in Vitro Models

**DOI:** 10.1002/jcp.25282

**Published:** 2015-12-28

**Authors:** Katherine A. Staines, Matt Prideaux, Steve Allen, David J. Buttle, Andrew A. Pitsillides, Colin Farquharson

**Affiliations:** ^1^ Roslin Institute and R(D)SVSThe University of EdinburghEaster BushMidlothianUnited Kingdom; ^2^ The University of AdelaideNorth TerraceAdelaideAustralia; ^3^ Royal Veterinary CollegeRoyal College StreetLondonUnited Kingdom; ^4^ Department of Infection and ImmunityThe University of Sheffield Medical School, Beech Hill RoadSheffieldUnited Kingdom

## Abstract

The transmembrane glycoprotein E11 is considered critical in early osteoblast–osteocyte transitions (osteocytogenesis), however its function and regulatory mechanisms are still unknown. Using the late osteoblast MLO‐A5 cell line we reveal increased E11 protein/mRNA expression (*P* < 0.001) concomitant with extensive osteocyte dendrite formation and matrix mineralization (*P* < 0.001). Transfection with E11 significantly increased mRNA levels (*P* < 0.001), but immunoblotting failed to detect any correlative increases in E11 protein levels, suggestive of post‐translational degradation. We found that exogenous treatment of MLO‐A5 and osteocytic IDG‐SW3 cells with 10 μM ALLN (calpain and proteasome inhibitor) stabilized E11 protein levels and induced a profound increase in osteocytic dendrite formation (*P* < 0.001). Treatment with other calpain inhibitors failed to promote similar osteocytogenic changes, suggesting that these effects of ALLN rely upon its proteasome inhibitor actions. Accordingly we found that proteasome‐selective inhibitors (MG132/lactacystin/ Bortezomib/Withaferin‐A) produced similar dose‐dependent increases in E11 protein levels in MLO‐A5 and primary osteoblast cells. This proteasomal targeting was confirmed by immunoprecipitation of ubiquitinylated proteins, which included E11, and by increased levels of ubiquitinylated E11 protein upon addition of the proteasome inhibitors MG132/Bortezomib. Activation of RhoA, the small GTPase, was found to be increased concomitant with the peak in E11 levels and its downstream signaling was also observed to promote MLO‐A5 cell dendrite formation. Our data indicate that a mechanism reliant upon blockade of proteasome‐mediated E11 destabilization contributes to osteocytogenesis and that this may involve downstream targeting of RhoA. This work adds to our mechanistic understanding of the factors regulating bone homeostasis, which may lead to future therapeutic approaches. J. Cell. Physiol. 231: 1392–1404, 2016. © 2015 The Authors. *Journal of Cellular Physiology* published by Wiley Periodicals, Inc.

The terminally‐differentiated, osteoblast‐derived osteocytes are the most numerous of all the cells within bone and are essential to bone structure and function (Dallas et al., 2013). Most original paradigms had proposed that osteocytes are passive in their formation (Skerry et al., 1989; Nefussi et al., 1991; Palumbo et al., 2004). Recent evidence, however, now indicates that initial “embedding” involves their active contribution with vital genetic and dramatic morphological transformations; not simply entrapment of defunct osteoblasts (Holmbeck et al., 2005). Along with concomitant dendrite formation, this creates bone's osteocyte‐canalicular network, which is now known to orchestrate bone remodelling (Bonewald, 2002, 2007, 2012). Compelling evidence for this “orchestrator” function comes from the discovery that osteocytes, deep in calcified bone, produce sclerostin, a Wnt inhibitor and potent negative modulator of bone formation (Balemans et al., 2001; Li et al., 2009; Staines et al., 2012b). Furthermore, it has been more recently shown that osteocytes can also communicate with bone‐resorbing osteoclast cells through RANKL expression (Nakashima and Takayanagi, 2011; Xiong et al., 2011).

Although it is well known that osteocytes are derived from osteoblasts, the mechanisms which govern this transition (osteocytogenesis) are yet to be elucidated. Many different genes have been suggested to influence osteocytogenesis, one of which encodes for the transmembrane glycoprotein E11. Although specific for osteocytes in bone, E11 is also widely expressed in many tissues throughout the body, such as the kidney and lung. It therefore has several names (podoplanin, gp38, T1 alpha, OTS‐8 among others) depending on its location and the species from which it was first isolated. E11 was the name given to the protein isolated from rat osteocytes by Wetterwald et al., (Wetterwald et al., 1996) and is therefore the common name used to describe this protein in relation to bone. The protein itself is a hydrophobic, mucin‐like, transmembrane glycoprotein, which can undergo post‐translational modification (via O‐glycosylation) leading to the production of different glycoforms. E11 is up‐regulated by hypoxia in the lung (Cao et al., 2003); IL‐3 and PROX‐1 in the lymphatic system (Hong et al., 2002; Groger et al., 2004) and TGF‐β in fibrosarcoma cells (Suzuki et al., 2005). The localisation of E11 in early embedding‐osteocytes identified it as a factor which likely contributes during the vital, early stages of osteocyte differentiation (Nefussi et al., 1991; Barragan‐Adjemian et al., 2006; Zhang et al., 2006). However, few studies have been performed to investigate the functions of E11 in osteocytes. It is known that E11 mRNA expression in osteocytes is up‐regulated in response to mechanical strain in vivo (Zhang et al., 2006). It has also been shown that the growth of cytoplasmic processes, which is induced by fluid‐flow in MLO‐Y4 cells, is abrogated in cells pre‐treated with siRNA targeted against E11 (Zhang et al., 2006). Over‐expression of E11 in ROS 17/2.6 osteoblast‐like cells led to the formation of long processes potentially via activation of the small GTPase, RhoA which acts through its downstream effector kinase ROCK to phosphorylate ezrin/moesin/radixin (ERM) and influence the actin cytoskeleton (Sprague et al., 1996; Martin‐Villar et al., 2014, 2006). These data, when taken collectively, suggest a key role for E11 in regulating the cytoskeletal changes associated with process formation and elongation. As the formation of such processes is a key feature of a differentiating osteocyte, this suggests an important functional role for the regulation of E11 during this mechanism, one which requires further examination.

In this study we have investigated the expression and regulation of E11 during osteocytogenesis. We found that E11 levels are regulated post‐translationally by proteasome degradation and that their preservation, by inhibition of this degradation, leads to the induction of an osteocyte‐like morphology in MLO‐A5 pre‐osteocytic cells, indicating the importance of E11 during osteocyte differentiation.

## Materials and Methods

### Animals

C57/BL6 mice were used in all experiments and kept in polypropylene cages, with light/dark 12‐h cycles, at 21 ± 2°C, and fed ad libitum with maintenance diet (Special Diet Services, Witham, UK). All experimental protocols were approved by Roslin Institute's Animal Users Committee and the animals were maintained in accordance with UK Home Office guidelines for the care and use of laboratory animals.

### Immunohistochemistry

Tibiae were dissected, fixed in 4% paraformaldehyde (PFA) for 24 h before being decalcified in 10% ethylenediaminetetraacetic acid (EDTA) pH 7.4 for approximately 3 weeks at 4°C with regular changes. Tissues were dehydrated and embedded in paraffin wax, using standard procedures, after which they were sectioned at 6 µm. For immunohistochemical analysis, sections were dewaxed in xylene and rehydrated. Sections were incubated at 37°C for 30 min in 1 mg/ml trypsin for antigen demasking. Endogenous peroxidases were blocked by treatment with 3% H_2_O_2_ in methanol (Sigma, Dorset UK). E11 antibodies (R&D systems, Oxford UK) were used at a dilution of 1/100, and sclerostin antibodies (R&D systems) at 1/200 with appropriate controls used. The Vectastain ABC universal kit (Vector Laboratories, Peterborough) was used according to the manufacturer's instructions. The sections were dehydrated, counterstained with haematoxylin and mounted in DePeX.

### Primary osteoblast isolation

Primary calvarial osteoblasts were obtained from 4‐day‐old C57Bl/6 mice by sequential enzyme digestion of excised calvarial bones using a four‐step process as has previously been described (Staines et al., 2013; Orriss et al., 2014) [1 mg/ml collagenase type II in Hanks' balanced salt solution (HBSS) for 10 min; 1 mg/ml collagenase type II in HBSS for 30 min; 4 mM EDTA for 10 min; 1 mg/ml collagenase type II in HBSS for 30 min]. The first digest was discarded and the cells were re‐suspended in growth medium consisting of a‐MEM (Invitrogen, Paisley UK) supplemented with 10% (v/v) FCS (Invitrogen) and 1% gentamycin (Invitrogen). Osteoblasts were seeded at a density of 1 × 10^4^ cells/cm^2^ and grown to confluency before being administered with proteasome inhibitors.

### MLO‐A5 cell culture

The murine MLO‐A5 pre‐osteocyte cell line was obtained from Dr. Lynda Bonewald (University of Missouri‐Kansas City). Cells were plated at 35 × 10^3^ cells/cm^2^ in multi‐well plates and cultured in α‐MEM medium supplemented with 5% (v/v) FBS, 5% (v/v) calf serum (Invitrogen) and 50 μg/ml gentamicin (Invitrogen) at 37°C with 5% CO_2_. At confluence (day 0), the medium was changed to α‐MEM supplemented with 10% (v/v) FBS, 50 µg/ml gentamicin, 5 mM β‐glycerol 2‐phosphate disodium salt hydrate (βGP, Sigma) and 100 μg/ml ascorbic acid (Sigma) in order to promote differentiation and matrix mineralization. The MLO‐A5 cells were incubated at 37°C in a humidified atmosphere containing 5% CO_2_ and the medium was changed every 2–3 days.

### ATDC5 cell culture

Chondrogenic murine ATDC5 cells (RIKEN cell bank, Ibaraki, Japan) were utilized as a well‐established model of chondrocyte matrix mineralization with previous studies detailing their chondrogenic differentiation and subsequent mineralization (Newton et al., 2012; Staines et al., 2012a). Cells were cultured in differentiation medium (DMEM/F‐12 (1:1) with GlutaMAX I containing 5% FBS, 1% insulin transferrin and selenium, 1% sodium pyruvate and 0.5% gentamicin [Invitrogen]) at a density of 6,000 cells/cm^2^. 10 mM βGP and 50 µg/ml ascorbic acid were added once the cells had reached confluence. Cells were incubated in a humidified atmosphere (37°C, 5% CO_2_) and the medium was changed every 2–3 days.

### IDG‐SW3 cell culture

The murine IDG‐SW3 osteocytic cell line was obtained from Dr. Lynda Bonewald (University of Missouri‐Kansas City). Cells were plated at 1 × 10^4^ cells/cm^2^ in multi‐well plates and cultured as previously described (Woo et al., 2011). Inhibitors were added to cultures at day 0 (confluency).

### Over‐expression of E11 by MLO‐A5 transfection

E11 was amplified from MLO‐A5 cDNA‐ using primers designed to flank the E11 open reading frame (forward 5′‐AGCTCGTTTAGTGAACCGTCAG‐3′, reverse 5′‐CCACTTGTGTAGCGCCAAGT‐3′) and the following cycling conditions: 35 cycles of 92°C for 30 sec, 61°C for 60 sec and 72°C for 60 sec. The polymerase chain reaction (PCR) product was run on a 1% agarose gel, purified and ligated into the pCR2.1‐TOPO cloning vector (Invitrogen) according to the manufacturer's instructions. Individual clones of transformed XL1‐Blue *E. coli* (Stratagene, Santa Clara) were isolated from agar plates and the plasmid DNA sequenced to identify those containing the E11 insert (Genepool, University of Edinburgh). The insert was removed from the plasmid using *EcoRI*, ligated into the pLVX‐puro vector (Clontech, CA) and used to transform *E. coli*. Plasmids were screened by restriction digest with *XhoI* to identify clones which contained the insert in the correct orientation and plasmid DNA was amplified and extracted using the EndoFree Maxiprep kit (Qiagen, Sussex, UK). MLO‐A5 and ATDC5 cells were transfected with E11‐ and empty vector‐containing plasmids using Fugene HD transfection reagent (Roche; according to manufacturer's instructions). Transfections were performed in triplicate and transfected cells were positively selected in media supplemented with 2.5 µg/ml puromycin.

### Effects of cysteine protease inhibitors on MLO‐A5 cells

The function of cysteine proteinases in controlling MLO‐A5 E11 protein levels and morphology was examined using varying concentrations (0–100 μM) of the protease inhibitors, calpastatin, calpeptin, acetyl‐l‐leucyl‐l‐leucyl‐l‐norleucinal (ALLN) (Calbiochem, CA), 2S,3S‐*trans*‐(ethoxycarbonyloxirane‐2‐carbonyl)‐L‐leucine‐(3‐methylbutyl) amide (E64d) (Cayman Chemicals, MI), and benzyloxycarbonyl‐Phe‐Ala‐fluoromethyl ketone (Z‐FA‐FMK) (Santa Cruz Biotechnology, CA). Briefly, confluent MLO‐A5 cells were treated with media supplemented with the aforementioned inhibitors at a range of concentrations, and then incubated at 37°C for a further 24 h. Thereafter, protein was extracted from monolayers, as described below, for the determination of E11 protein expression by western blotting. To examine the effects of ALLN on cell morphology, MLO‐A5 cells were seeded at 5 × 10^4^ per well in 6 well plates and cultured overnight at 37°C before supplementation with 15 µM ALLN for an additional 48 h. Changes in cell morphology were monitored using brightfield microscopy and quantified by counting the number of cells (n = 100 randomly selected) in triplicate cultures, displaying projections longer than 25 µm.

### Effects of proteasome inhibitors on MLO‐A5 and primary osteoblast cells

Confluent MLO‐A5 cell cultures were treated with media supplemented with 0–10 μM N‐(benzyloxycarbonyl)‐Leu‐Leu‐Leucinal (MG132), lactacystin (Cayman Chemicals), Bortezomib (Velcade®) (Santa Cruz), and Withaferin‐A (Abcam, Cambridge UK) and then incubated at 37°C for 24 h. Confluent primary cell cultures were treated with media supplemented with ALLN, MG132, and lactacystin. Thereafter, protein/RNA was extracted from monolayers, as described below, for the determination of E11 protein/mRNA expression.

### Effects on RhoA/ROCK pathway in MLO‐A5 cells

MLO‐A5 cells were cultured in the presence of increasing concentrations of the ROCK inhibitors, Y27632 (0–50 µM; Cambridge Bioscience) or the more potent H‐1152, (0–50 µM; Cambridge Bioscience) and protein expression levels examined by western blotting. MLO‐A5 cells were treated with H‐1152 in the presence/absence of ALLN for 48 h. Changes in cell morphology were monitored using brightfield microscopy and quantified by counting the number of cells (among n = 100 randomly selected) in triplicate cultures, displaying projections longer than 25 µm.

### RNA extraction and quantitative real‐time PCR (RT‐qPCR)

Total RNA was extracted from MLO‐A5 cells using Tri‐Reagent according to the manufacturer's instructions. RNA samples were reverse‐transcribed into cDNA using Superscipt II reverse transcriptase (Invitrogen) according to the manufacturer's instructions. RT‐qPCR was carried out in a Stratagene Mx3000P cycler with each reaction containing 50ng template DNA, 250nM forward and reverse primers (Primer Design, Southampton UK) (*E11*: F‐ CTAACCACCACTCCCACTT, R‐ CCAATAGACTCCAACCTGAAGA; *Gapdh*: sequences not available) and PrecisionPlus Mastermix (Primer Design). The Ct values for the samples were normalised to that of *Gapdh* and the relative expression was calculated using the ΔΔCt method (Livak and Schmittgen, 2001). The amplification efficiencies of all the primers were between 90–100%.

### Western blotting

Protein lysates were extracted and concentrations were determined using the DC assay (Bio‐Rad, Hemel Hempstead, UK) and 15 µg of protein was separated using a 10% bis‐tris gel and then transferred to a nitrocellulose membrane and probed with goat anti‐mouse E11 (1:1000, R&D Systems) and HRP‐linked rabbit anti‐goat secondary antibody (1:3,000, Dako, Cambridge, UK); or rabbit anti‐mouse proteasome subunit beta type‐5 (PSMB5) (1:1,000, Abcam) and HRP‐linked goat anti‐rabbit secondary antibody (1:3,000, Dako); or rabbit anti‐mouse RhoA (1:1,000, Cell signaling) and HRP‐linked goat anti‐rabbit secondary antibody (1:3,000, Dako) diluted in 5% non‐fat milk. Rabbit anti‐mouse ERM and phospho‐ERM (1:1000, Invitrogen) antibodies and HRP‐linked goat anti‐rabbit secondary antibody (1:3,000, Dako) were diluted in 3% bovine serum albumin.

Immune complexes were visualised by chemiluminescence using the ECL detection kit and ECL film (GE Healthcare, Amersham, UK). HRP‐conjugated anti β‐actin antibody (1:25,000, Sigma) was used as a loading control.

### RhoA activity assay

Protein was extracted from MLO‐A5 cultures and RhoA activation measured by the GLISA assay (Cytoskeleton, Denver), which specifically recognises active GTP‐bound RhoA. The quantity of GTP‐bound RhoA was then measured by luminometry and expressed in relative light units (RLU).

### Proteasome activity

Protein lysates were extracted from MLO‐A5 cells in 0.5% NP40 at days 0, 3, 7, 10, and 14 of culture. A proteasome activity kit (Abcam), which takes advantage of the chymotrypsin‐like activity of the proteasome, was used according to the manufacturer's instructions. The kit utilises an aminomethylcoumarin (AMC)‐tagged peptide substrate which releases free, highly fluorescent AMC in the presence of proteolytic activity. Fluorescence was measured with a microtiter plate reader in real time for 30–60 min at Ex/Em = 350/440 nm at 37°C. Proteasome activity was subsequently calculated according to the manufacturer's instructions.

### Isolation and analysis of ubiquitinylated proteins

Protein lysates were extracted from MLO‐A5 cells in 50 mM HEPES, pH 7.6, 1 mM DTT and samples were diluted to 25 µg/100 µl. Ubiquitinylated proteins were extracted using the UbiQapture^®^‐Q kit (Enzo Life Sciences, Exeter, UK) according to the manufacturer's instructions. Extracted ubiquitinylated proteins (bound fraction), as well as non‐ubiquitinylated proteins (unbound fraction), were separated using a 10% bis‐tris gel and western blotting was conducted for E11 protein as detailed above.

### Statistical analysis

Data are expressed as the mean ± standard error of the mean (S.E.M) of at least three replicates per experiment. Statistical analysis was performed by Student's t‐test, one way analysis of variance (ANOVA) or a suitable non‐parametric test. *P* < 0.05 was considered to be significant and noted as *P values of <0.01 and <0.001 were noted as “**” and “***” respectively.

## Results

### E11 expression increases during osteocyte differentiation

**Figure 1:**
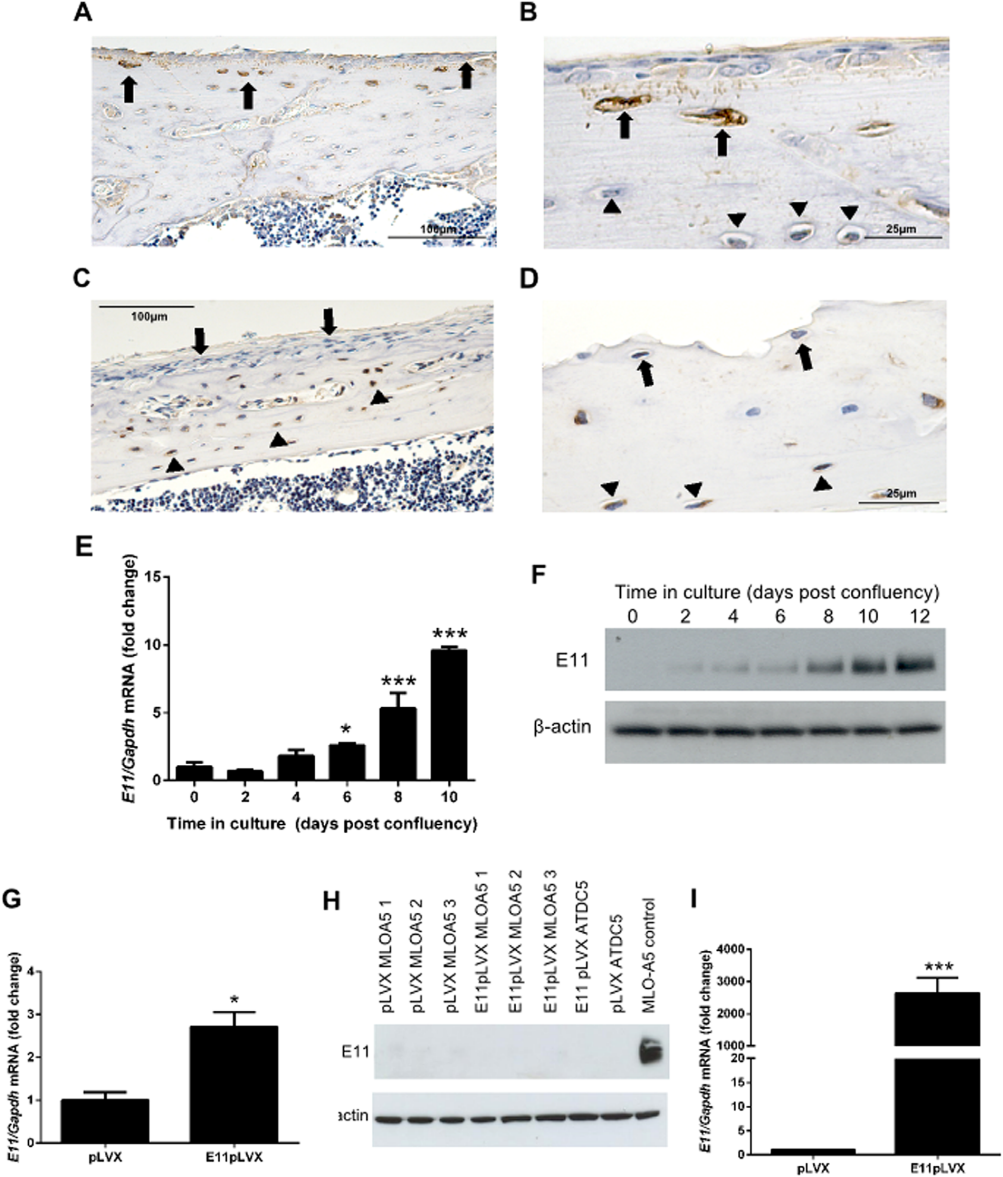
E11 expression increases during osteocyte differentiation. (A) Immunohistochemical staining for E11 protein expression in 8‐week‐old wild‐type mouse tibia. (B) Expression was localised to young osteocytes (arrows) and their processes (arrows). Mature osteocytes were devoid of E11 expression (arrowhead, B). (C and D) Labelling for sclerostin expression was observed in mature osteocytes (arrowheads) but not in the young osteocytes close to the periosteum (arrows). Scale bars are 100 µm (A and C), and 25 µm (B and D). Results are representative of five individual mice. (E) RT‐qPCR analysis of *E11* mRNA expression in mineralizing MLO‐A5 cells over a 10 day culture period. Data are represented as mean ± S.E.M of three individual cultures. *P < 0.05, ***P < 0.001 in comparison to day 0. (F) Western blotting for E11 protein expression in MLO‐A5 cells over a 12 day culture period. (G) RT‐qPCR for *E11* mRNA in MLO‐A5 cells transfected with E11 overexpressing vectors (E11pLVX) and control cultures (pLVX). (H) Western blotting of E11 protein upon overexpression in MLO‐A5 cells (E11pLVX) at day 0 in comparison to control cultures (pLVX). Also displayed is E11pLVX and pLVX in the chondrocytic ATDC5 cell line, and in MLO‐A5 control cultures (i.e. non‐transfected cells) at day 15 of culture. (I) *E11* mRNA expression by RT‐qPCR in E11pLVX and pLVX transfected ATDC5 cells. Data are represented as mean ± S.E.M of three individual cultures. *P < 0.05, or ***P < 0.001, in comparison to control cultures (pLVX) at day 0.

Our previous studies indicated that extracellular matrix (ECM) mineralization promotes E11 expression and drives osteocytic differentiation (Prideaux et al., 2012). To more closely examine the relationship between E11 expression and osteocytogenesis, we examined spatial patterns of E11 protein expression during various stages of osteoblast‐to‐osteocyte transition in vivo. Immunohistochemical examination of tibia sections from 8‐week‐old mice disclosed that E11 protein was detected in young, recently transitioned osteocytes close to the periosteum (Fig. 1A and B). We also observed that osteocyte processes connecting the young osteocytes to surface osteoblasts were labelled positively for E11 protein whereas osteoblasts on the bone surface and more mature osteocytes deeper within the cortical bone matrix were devoid of labelling for E11 protein (Fig. 1A and B). Consistent with this, immunolabelling for sclerostin, a known marker of the late osteocyte phenotype revealed expression in the mature osteocytes which was not observed in the young osteocytes close to the periosteum (Fig. 1C and D).

Whilst these observations further strengthen the link between E11 expression and the transition from osteoblast into the osteocyte phenotype, they do not inform their dynamic relationship. We therefore examined E11 mRNA and protein expression in MLO‐A5 cells (late osteoblast/pre‐osteocytic cells) cultured under mineralizing conditions to promote their osteocytic differentiation. These studies revealed that the levels of E11 mRNA increased significantly between days 0 and 6 (*P* < 0.05), and continued to do so throughout the culture period to day 10 of culture (*P* < 0.001) (Fig. 1E). Similar increases in E11 protein expression were also observed (Fig. 1F). These results are consistent with our previous data highlighting the potent up‐regulation of E11 during the osteoblast‐to‐osteocyte transition (Prideaux et al., 2012).

### Lack of E11 protein over‐expression despite elevated mRNA levels in transfected MLO‐A5 cells

Such spatial and temporal association between increases in E11 expression and acquisition of osteocyte phenotype does not, however, necessarily indicate a causal relationship. To provide more direct functional evidence that E11 expression might control the phenotypic changes taking place during osteoblast‐to‐osteocyte transition, we generated an in vitro system in which E11 gain‐of‐function could be engendered in MLO‐A5 cells by transfection, and subsequent mRNA and protein levels assessed by RT‐qPCR and western blotting, respectively.

We found that transfection of MLO‐A5 cells with E11‐containing plasmids produced 2.5‐fold increases in *E11* mRNA levels when compared to cells transfected with empty vector (Fig. 1G). We subsequently sought to confirm such E11 gain‐of‐function by measuring increases in E11 protein levels in these cells. Intriguingly, western blotting failed to detect any significant elevation in E11 protein levels in MLO‐A5 cells that were transfected with the E11‐containing plasmid (Fig. 1H). To examine the possibility that high endogenous E11 levels may limit the transfection‐related increases in expression in MLO‐A5 cells, we also examined E11 transfection efficiency in chondrogenic ATDC5 cells in which E11 is not constitutively expressed. Western blotting similarly failed to detect any significant expression of E11 protein in ATDC5 cells transfected with the E11‐containing plasmid (Fig. 1H), despite substantial increases in mRNA expression in these transfected cells (Fig. 1I). These data indicate lack of correlative increases in E11 mRNA and protein levels and therefore suggest that E11 protein is inherently unstable or selectively targeted, post‐translationally, for degradation.

### E11 protein is post‐translationally regulated in MLO‐A5 cells and this limits acquisition of osteocyte phenotype achieved via E11

**Figure 2:**
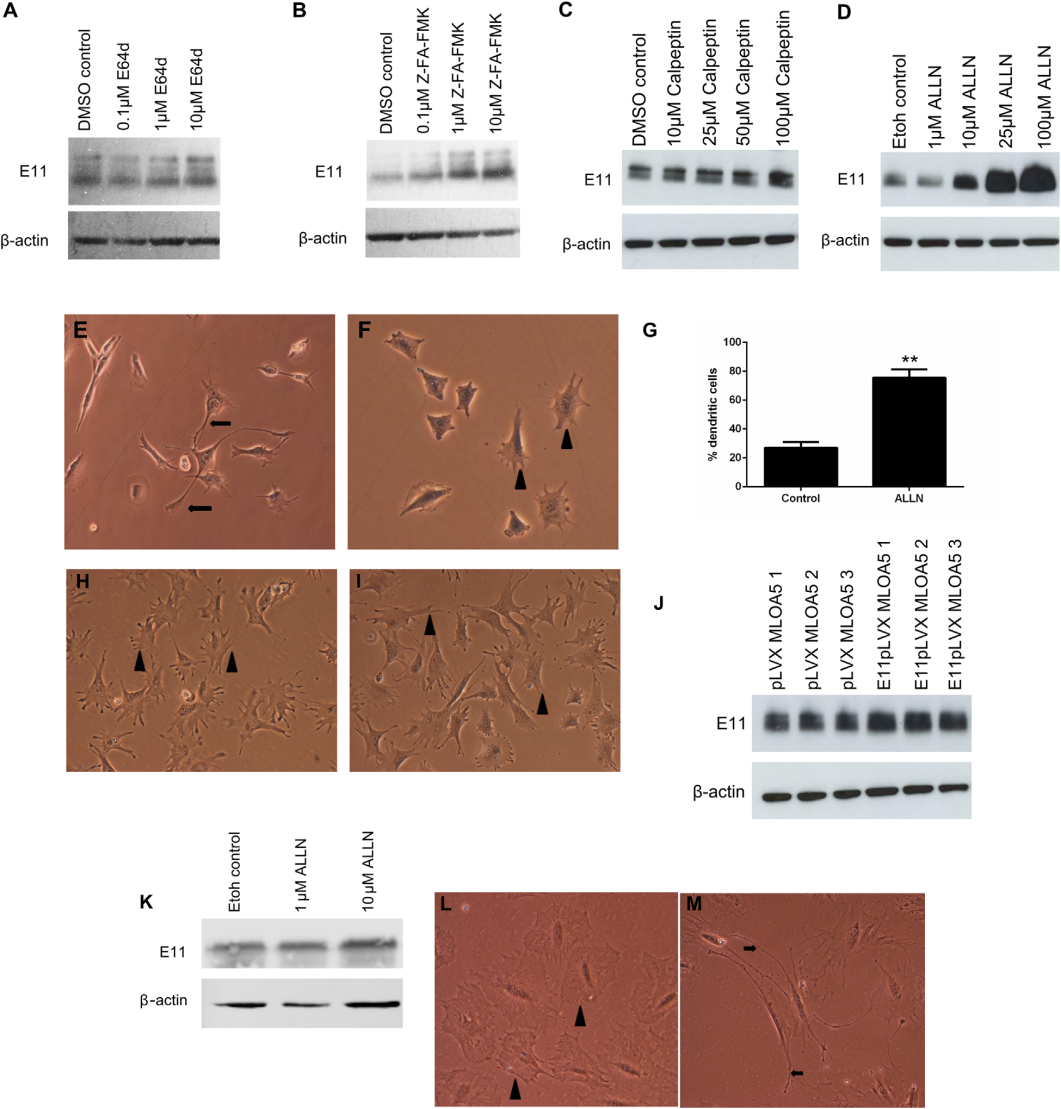
Western blotting of E11 protein (∼38 kDa) expression in MLO‐A5 cells treated with increasing concentrations of (A) E64d, (B) Z‐FA‐FMK, (C) Calpeptin, (D) ALLN, for 24 h. β‐actin was used as a loading control. Effect of ALLN treatment on MLO‐A5 cell morphology after 48 h with (E) 10 µM ALLN and (F) 0 µM ALLN treatment. Control cultures display numerous short cell projections (arrowheads, F) in comparison to treated cells which display multiple long, thin cytoplasmic cellular projections (arrows, E). (G) The percentage of MLO‐A5 cells expressing an elongated dendritic‐like morphology after treatment with ALLN in comparison to control cultures. Data are represented as mean ± S.E.M of three individual experiments. **P < 0.01 in comparison to control cultures. Effect of calpeptin treatment on MLO‐A5 cell morphology after 48 h with (H) 10 µM calpeptin (I) 10 µM calpeptin; both control and calpeptin‐treated cells displayed short, cell projections (arrowheads). (J) Overexpression of E11 in MLO‐A5 cells upon addition of 15 µM ALLN (E11pLVX) at day 0 for 24 h, in comparison to control cultures treated with 15 µM ALLN (pLVX). (K) Western blotting of E11 protein (∼38 kDa) expression in IDG‐SW3 cells treated with increasing concentrations of ALLN. Effect of ALLN treatment on IDG‐SW3 cell morphology after 48 h 10 µM ALLN treatment. Control cultures display numerous short cell projections (arrowheads, L) in comparison to treated cells which display multiple long, thin cytoplasmic cellular projections (arrows, M). β‐actin was used as a loading control.

It has previously been suggested that E11 is degraded by the calpain family of cysteine proteinases (Martin‐Villar et al., 2009). Therefore, to investigate whether the E11 protein is similarly degraded by this family of proteases in MLO‐A5 cells, cultures were supplemented with varying concentrations of the calpain inhibitors, calpeptin, E64d, Z‐FA‐FMK, and ALLN. These studies revealed that increasing concentrations of E64d failed to increase E11 protein expression in MLO‐A5 osteoblast cells (Fig. 2A). Similarly, only modest increases in E11 protein levels were seen in MLO‐A5 cells treated with Z‐FA‐FMK and calpeptin (Fig. 2B and C). However, in contrast, ALLN produced stark and dose‐dependent increases in E11 protein levels (Fig. 2D).

Furthermore, when we examined changes in MLO‐A5 cell morphology we found that in addition to producing marked increases in levels of E11 protein, ALLN also induced a profound elongated dendritic‐like morphology in MLO‐A5 cells, with some cell processes extending from the cell body to over 100 μm in length, consistent with the dendritic processes seen in osteocytes (Fig. 2E). This is in contrast to MLO‐A5 cells supplemented with vehicle alone (ethanol) which displayed a standard morphology, with numerous small cellular projections (Fig. 2F). Similarly, cells treated with 10 µM calpeptin did not exhibit any differences in morphology in comparison to control cultures (Fig. 2H and I). Quantification of cells displaying an elongated dendritic‐like morphology showed that their percentage among ALLN‐treated cells was significantly increased (77%, *P* < 0.001***) compared to control cultures (27%) (Fig. 2G). These data suggest that inhibition of E11 breakdown increases acquisition of the osteocyte phenotype.

Consistent with the possibility that E11 is targeted for post‐translational degradation by mechanisms sensitive to ALLN, addition of 15 μM ALLN to MLO‐A5 cells transfected with empty vector now resulted in strong and robust expression of E11 protein (Fig. 2J). Relatively modest further increases in E11 protein levels were observed in MLO‐A5 cells transfected with the E11‐containing vector, consistent with the previously detected 2.5‐fold increase in *E11* mRNA expression (Fig. 1G). Treatment of the murine osteocytic cell line IDG‐SW3 (Woo et al., 2011), with ALLN also showed modestly increased E11 protein expression upon western blotting with low concentrations of ALLN (Fig. 2K) and extensive dendrite formation (Fig. 2L and M).

Together these data suggest that E11 protein is subject to active, constitutive degradation via ALLN‐sensitive pathways in MLO‐A5 and IDG‐SW3 cells, and that blockade of these pathways correlates with enhanced osteocyte cell differentiation.

### Calpain 1 and 2 are unlikely to be involved in regulating E11 protein expression during MLO‐A5 differentiation

Based on published data demonstrating that calpains can degrade E11 *in vitro*, we speculated that the observed potent effects of ALLN on E11 protein levels in MLO‐A5 cells were likely dependent on the inhibition of calpain activity (Martin‐Villar et al., 2009).

**Figure 3:**
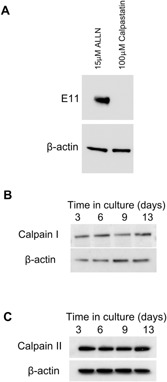
(A) Western blotting of E11 protein (∼38 kDa) expression in MLO‐A5 cells treated with 15 μM ALLN and 100 μM calpastatin. Western blotting of (B) calpain I and (C) calpain II protein in mineralising MLO‐A5 cells over a 15‐day culture period. β‐actin was used as a loading control.

We explored this possibility by monitoring E11 protein levels in MLO‐A5 cells following addition of the physiological calpain‐specific inhibitor, calpastatin and found that, unlike ALLN, this failed to promote increases in E11 protein levels (Fig. 3A). Western blotting of lysates from differentiating MLO‐A5 cells also indicated that the protein expression levels of calpain I and II were unchanged at all time‐points (Fig. 3B and C). Together, with our previous data showing that E11 stability was influenced by only the very highest calpeptin concentrations (Fig. 2C), these observations suggest that calpains are unlikely to be responsible for E11 degradation and that the effects observed upon addition of ALLN are likely via alternative mechanisms.

### Levels of E11 are regulated via proteasome pathways during osteocytogenesis in MLO‐A5 cells

As well as having inhibitory effects on calpains and cathepsins, ALLN is also capable of exerting inhibition of proteasome‐mediated protein degradation, as are calpeptin and Z‐FA‐FMK which also stimulated modest E11 protein increases (Giguere and Schnellmann, 2008). Therefore to examine whether the proteolytic mechanism underpinning E11 stability resides within the proteasome, we sought to determine the effect of the specific proteasome inhibitors lactacystin and MG132 on MLO‐A5 E11 protein expression.

**Figure 4:**
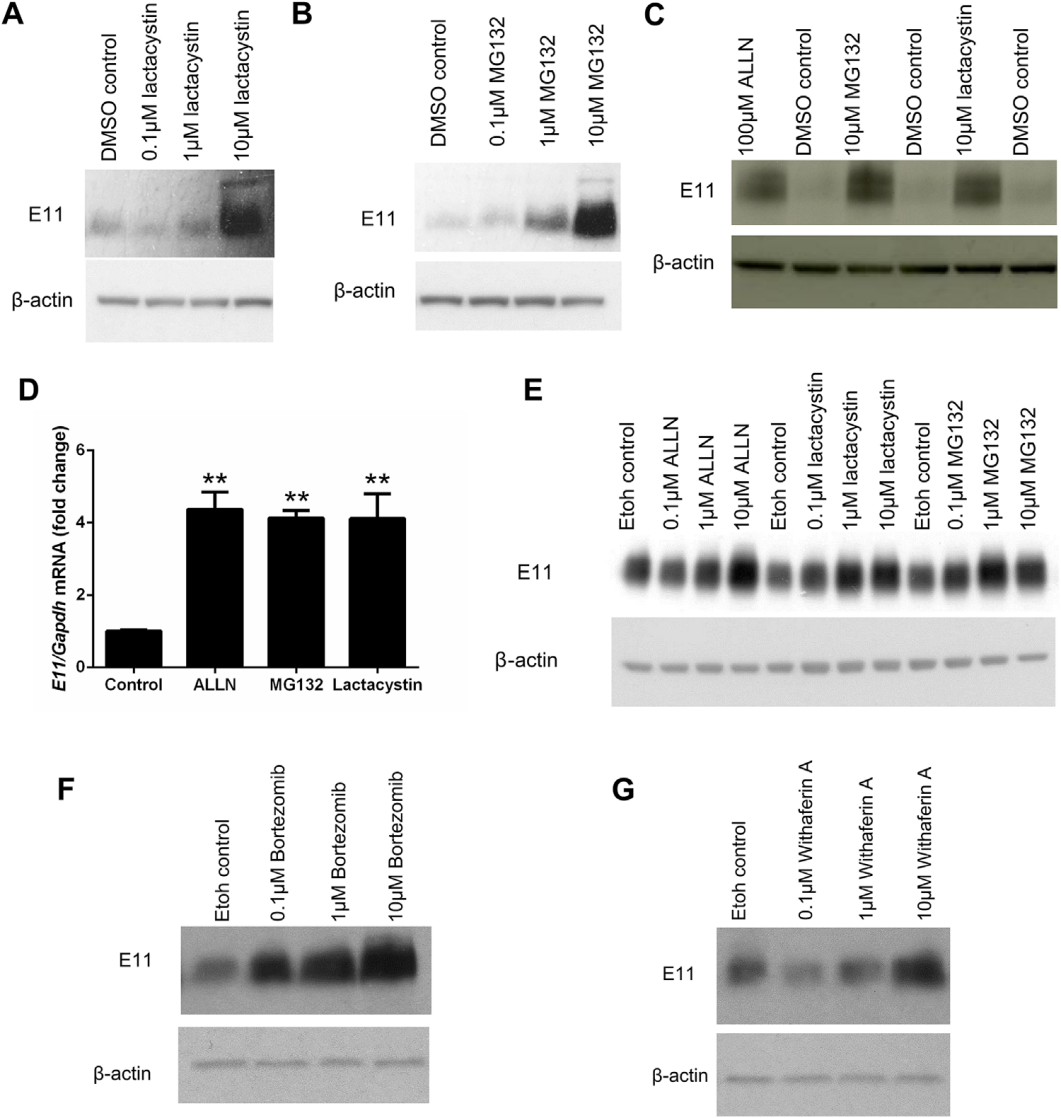
Western blotting of E11 protein (∼38 kDa) expression in MLO‐A5 cells treated with 0 μM, 0.1 μM, 1 μM, and 10 μM of (A) Lactacystin (B) MG132 for 24 h. β‐actin was used as a loading control. (C) Western blotting of E11 protein (∼38 kDa) expression in MLO‐A5 cells treated with 100 μM ALLN, 10 μM MG132 and 10 μM lactacystin for 24 h. β‐actin was used as a loading control. (D) RT‐qPCR analysis of *E11* mRNA expression in MLO‐A5 cultures following addition of 100 μM ALLN, 10 μM MG132, and 10 μM lactacystin for 24 h. Data are represented as mean ± S.E.M of three individual cultures. **P < 0.01 in comparison to control cultures. (E) Western blotting of E11 protein (∼38 kDa) expression in primary osteoblast cells treated with 0, 0.1, 1, and 10 μM of ALLN, lactacystin and MG132 for 24 h. β‐actin was used as a loading control. Western blotting of E11 protein (∼38 kDa) expression in MLO‐A5 cells treated with 0, 0.1, 1, and 10 μM of (F) Bortezomib (G) Withaferin A for 24 h. β‐actin was used as a loading control.

Addition of both MG132 and lactacystin resulted in a dramatic increase in E11 protein expression levels within 24 h. Enhanced E11 protein levels were noticeable at concentrations of lactacystin and MG132 as low as 10 μM and 1 μM, respectively (Fig. 4A and B), comparable with those achieved upon 100 μM ALLN treatment (Fig. 4C). Similar increases in *E11* mRNA were observed in MLO‐A5 cells upon addition of the inhibitors ALLN, MG132, and lactacystin for 24 h (Fig. 4D). However, increased transcription upon treatment with proteasome inhibitors has previously been reported in differing cell types suggesting an additional mechanism by which the proteasome may function in osteocytes (Butler et al., 2009; Rockwell and Qureshi, 2010).

Increases in E11 protein expression were also observed in primary osteoblast cells treated with increasing concentrations of ALLN, MG132, and lactacystin (Fig. 4E) once they had reached confluency. The expression of E11 in these cultures at this early time point has been reported in previous studies by us and others (Zhang et al., 2006; Prideaux et al., 2012). Furthermore, addition of the proteasome inhibitor Bortezomib also induced a dose‐dependent increase in E11 protein expression after 24 h (Fig. 4F). Increases in E11 protein expression above control levels were also observed in MLO‐A5 cells upon addition of Withaferin A, a proteasome inhibitor isolated from *Withania somnifera* (Fig. 4G).

**Figure 5:**
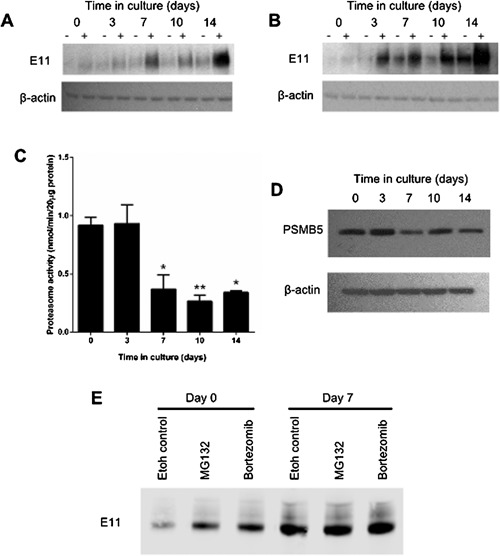
Western blotting of E11 protein (∼38 kDa) expression in MLO‐A5 cells treated with ± 10 μM of (A) MG132 (B) Bortezomib for 24 h prior to each time point over a 14 day culture period. β‐actin was used as a loading control. (C) Proteasome activity in MLO‐A5 cells over a 14 day culture period. Data are represented as mean ± S.E.M of three individual cultures. *P < 0.05, **P < 0.01 in comparison to day 0. (D) Western blotting of PSMB5 (∼25 kDa) expression in MLO‐A5 cells over a 14 day culture period. β‐actin was used as a loading control. (E) Immunoprecipitation of all ubiquitinylated proteins identified E11 to be expressed at both days 0 and 7 of MLO‐A5 cell culture, both ± addition of 10 μM MG132/Bortezomib. Data are represented as mean ± S.E.M.

These data strongly implicate proteasome degradation, rather than cysteine proteases in controlling E11 stability and hence levels. We next examined whether this process of E11 protein stabilization was active at all stages of osteoblast‐to‐osteocyte transition, by evaluating the effects of these selected proteasome inhibitors on E11 levels at various time points during MLO‐A5 cell osteocytic differentiation. Western blotting revealed that E11 protein is indeed increased by MG132 (Fig. 5A) and by Bortezomib (Fig. 5B) at all stages of MLO‐A5 cell differentiation.

The increased levels of E11 protein at osteoblast‐to‐osteocyte transition suggest an endogenous control of proteasome activity in these osteocyte cells. We therefore examined proteasome activity throughout MLO‐A5 cell differentiation and found it to be significantly decreased from day 7 of culture (*P* < 0.01 in comparison to day 0). Proteasome activity continued to be decreased at later stages of culture (*P* < 0.01 and *P* < 0.05 at days 10 and 14, respectively, in comparison to day 0) (Fig. 5C). Furthermore, our results revealed a trend towards decreased expression (from day 7 onwards) of PSMB5 protein, the major proteasome subunit, (Fig. 5D) with MLO‐A5 cell osteocytogenesis. Finally, to further confirm our findings we used immunoprecipitation to isolate all ubiquitinylated proteins and found E11 to be among the proteins expressed in the ubiquitinylated form at both days 0 and 7 of MLO‐A5 cell culture (Fig. 5E). Furthermore, as expected, ubiquitinylated E11 protein levels were increased upon addition of the proteasome inhibitors MG132 and Bortezomib at both these time points (Fig. 5E).

### E11 stabilization promotes osteocyte differentiation through RhoA

**Figure 6:**
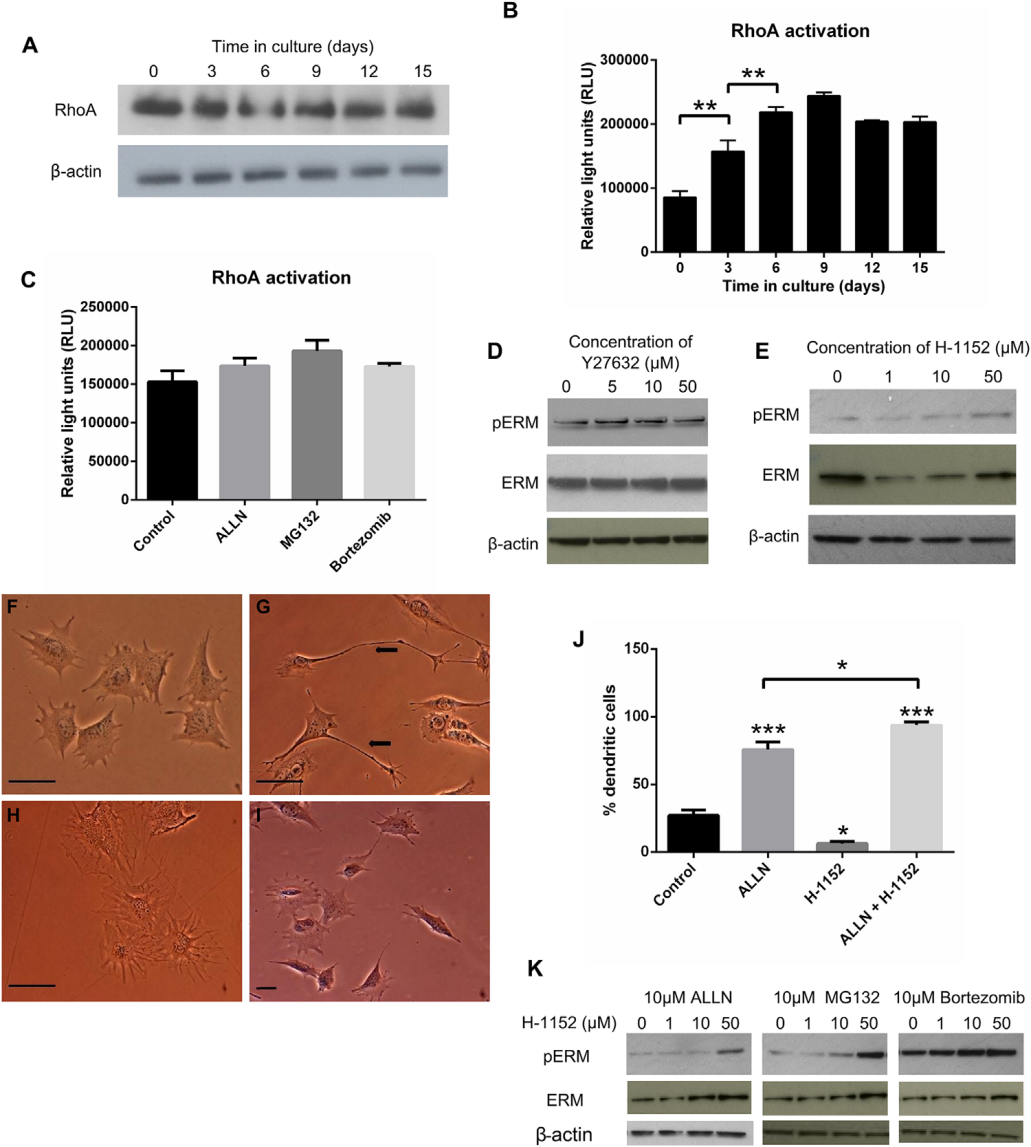
(A) Western blotting of RhoA protein expression in MLO‐A5 cells over a 15 days culture period. β‐actin was used as a loading control. (B) Quantification of GTP‐bound RhoA (RhoA activity) in MLO‐A5 cells over a 15 days culture period. Data are representative of two independent experiments. ***P < 0.001 in comparison to day 0. (C) Quantification of GTP‐bound RhoA (RhoA activity) in MLO‐A5 cells over treated with 10 μM ALLN, 10 μM MG132 and 10 μM Bortezomib for 24 h. Data are represented as mean ± S.E.M of three individual cultures in duplicate (D) Western blotting of phosphorylated ERM (∼80 kda) and total ERM (∼75 kDa) in MLO‐A5 cells cultured in the presence of 0, 5, 10, and 50 µM Y27632. β‐actin was used as a loading control. (E) Western blotting of phosphorylated ERM (∼80 kda) and total ERM (∼75kDa) in MLO‐A5 cells cultured in the presence of 0, 1, 10, and 50 µM H‐1152. β‐actin was used as a loading control. Effect of H‐1152 and ALLN treatment on MLO‐A5 cell morphology after 48 h with (F) vehicle treated, (G) 10 µM ALLN treatment (H) 10 µM H‐1152 (I) 10 µM H‐1152 and 10µM ALLN treatment. (J) The percentage of MLO‐A5 cells expressing an elongated dendritic‐like morphology after treatment with ALLN, H‐1152, and ALLN & H‐1152 in comparison to control cultures. Data are represented as mean ± S.E.M of three individual experiments. **P < 0.01, ***P < 0.001 in comparison to control cultures. (K) Western blotting of phosphorylated ERM (∼80 kda) and total ERM (∼75 kDa) in MLO‐A5 cells cultured in the presence of 0, 1, 10, and 50 µM H‐1152 with 10 µM ALLN or MG132 or Bortezomib. β‐actin was used as a loading control.

To investigate the mechanisms by which E11 stabilization promotes osteocyte differentiation, we examined the role of the RhoA/ROCK/ERM pathway. In MLO‐A5 cells, we found that RhoA levels were unchanged throughout the culture period (Fig. 6A). Interestingly, RhoA activation (GTP‐bound RhoA) was however increased in MLO‐A5 cells from day 3 of culture, concurrent with cell differentiation and matrix mineralization, in a pattern which mimicked E11 expression (Fig. 6B). Similarly, stabilization of E11 through the administration of proteasome inhibitors also increased RhoA activity (13–26% in comparison to control cultures) further suggesting that E11 acts to promote osteocytogenesis through this pathway.

To further interrogate the role of this pathway in E11‐mediated osteocytogenesis, we cultured MLO‐A5 cells in the presence of the ROCK inhibitor Y27632 (Martin‐Villar et al., 2006). Unexpectedly, increasing concentrations of Y27632 did not alter phosphorylation of the ROCK downstream target ERM, in MLO‐A5 cells (Fig. 6C), nor did the more potent ROCK inhibitor H‐1152 (Fig. 6D).

Examination of cell morphology in MLO‐A5 cells treated with H‐1152, revealed a flattened cell shape without discernible processes (Fig. 6G) in comparison to those treated with 10 µM ALLN (Fig. 6F) which, as before, showed marked elongated dendritic‐like cell morphology compared to vehicle‐treated controls (Fig. 6E). Intriguingly, MLO‐A5 cells treated with a combination of ALLN and H‐1152 in contrast showed very dramatic alterations in cell phenotype with strikingly long and elaborate processes (Fig. 6H); quantification confirmed significant increases compared to both control (*P* < 0.001) and ALLN treated cultures (*P* < 0.05) (Fig. 6I). Similarly, high H‐1152 (50 µM) concentrations combined with ALLN increased phosphorylation of ERM protein, in comparison to cultures treated with 10 µM ALLN alone (Fig. 6J). Similar results were observed with cells treated with both H‐1152 and 10 µM MG132 or 10 µM Bortezomib (Fig. 6J). Together with previous data these findings suggest that osteocytogenesis involves E11‐mediated RhoA activation that is dependent upon the inhibition of the proteasome in late, pre‐osteocytic, osteoblasts.

## Discussion

Our studies have shown for the first time that E11/podoplanin is a short‐lived protein and that mechanisms reliant on the blockade of proteasome‐mediated E11 destabilization contribute to the osteocytogenic recruitment of bone cells. Our data show the potent up‐regulation of E11 during the osteoblast‐to‐osteocyte transition, suggestive of E11 playing a critical role in early osteocyte differentiation as has previously been described by us, and others (Nefussi et al., 1991; Barragan‐Adjemian et al., 2006; Zhang et al., 2006; Prideaux et al., 2012 Dallas et al., 2013). We have previously shown that E11 is also intimately regulated by the ECM mineralization status (Prideaux et al., 2012). Here we have complemented this with clear evidence that induction of E11 protein expression in undifferentiated cells results in dendrite formation and elongation. The osteocyte process network is vital for maintaining cell viability and allowing the transfer of nutrients and waste products between osteocytes buried deep in the bone and the circulatory network (Bonewald, 2012). These results therefore suggest that E11 plays an important role during osteocyte formation. These data are consistent with a recent publication detailing the role of E11 in invadopodia stabilization and maturation in squamous cell carcinomas (Martin‐Villar et al., 2014).

To further delineate this mechanistic role, we examined the morphological effects of E11 gain‐of‐function in MLO‐A5 cells. Surprisingly, however, although we could show increases in *E11* mRNA in suitably transfected MLO‐A5 cells, this did not produce concomitant increases in E11 protein expression. This lack of correlation between E11 protein and mRNA levels was more pronounced in the ATDC5 chondrocyte cell line where basal E11 levels were negligible. We appreciate that as no E11 protein expression is observed in the empty‐vector transfected cultures at day 0, it may be viewed that there is no capability for the MLO‐A5 cells to overexpress E11 at this time point. However our data, combined with the lack of E11 protein in undifferentiated MLO‐A5 cells, which express *E11* mRNA, indicate profound post‐transcriptional and/or post‐translational regulation of E11 protein levels.

E11 has previously been shown to be a substrate for the neutral cysteine proteinase, calpain II, with many different tumour cell lines expressing high levels of *E11* mRNA but not E11 protein (Martin‐Villar et al., 2009). We report here that specific inhibition of calpain activity using the endogenous inhibitor calpastatin did not result in an accumulation of E11 protein in MLO‐A5 cells. An amplifying effect was observed, however, on both E11 protein levels and osteocyte dendrite formation using the inhibitor ALLN. This is strong, albeit indirect evidence that elevated levels of E11 are pivotal for the formation of osteocyte dendritic projections and extends previous studies in which dendrite formation was shown to be blocked by E11 siRNA (Zhang et al., 2006).

ALLN is a cell‐permeable inhibitor of calpains I and II, which is known to inhibit cathepsins B and L, and the proteasome. Here we identified the proteasome as the specific target of ALLN in MLO‐A5 cells through examination of the effects of a range of other proteases and proteasome inhibitors on E11 and ubiquitin levels. This result is contradictory to that observed by Martin‐Villar et al., in tumour cell lines whereby they used ALLN, MG132, calpeptin and lactacystin and showed that calpeptin displayed the greatest effect, whilst ALLN and MG132 induced only modest increases in E11 protein expression (Martin‐Villar et al., 2009). The very elegant studies of Martin‐Villar et al., also report that lactacystin had no effect on E11 expression. On this basis, the authors infer that calpains regulate E11 expression in this cell line. Whilst calpeptin is generally considered as a calpain inhibitor, like ALLN and MG132 it also has inhibitory effects on the proteasome (Giguere and Schnellmann, 2008) and as our differing conclusions may be due to different considerations of inhibitor specificity and/or suggests that E11 may be subject to differential regulatory mechanisms dependent upon cell type (Martin‐Villar et al., 2009). It also highlights that E11 stability may vary in different circumstances and that this is an important consideration should it ever be identified as a therapeutic target. Nevertheless, we observed similar results to our MLO‐A5 data in both primary calvaria osteoblasts and the late osteoblast‐osteocytic IDG‐SW3 cell line, thus providing further evidence that the proteasome plays the critical role in E11 regulation in osteoblasts.

The ubiquitin‐proteasome system functions to degrade regulatory and abnormal proteins. As such, proteasome activity, conferred by six catalytic active sites which have chymotrypsin‐like, trypsin‐like, and caspase‐like activities, is tightly regulated and attuned to cellular requirements. Here we reveal decreased proteasome activity upon MLO‐A5 osteocyte cell differentiation. Indeed, it is already well known that the ubiquitin‐proteasome pathway exerts exquisite control of osteoblast differentiation, with administration of proteasome inhibitors increasing bone volume and bone formation rates (Garrett et al., 2003). As such, it is possible that our observed decreases in proteasome activity during MLO‐A5 cell osteocytogenesis may explain the concomitant increases in E11 protein expression, and for the first time point to a critical role for the proteasome in regulating osteocyte differentiation. Whether stabilizing E11 increases sclerostin levels in concordance with promotion of osteocytogenesis is an interesting consideration, however, not one examined here due to the negligible levels of sclerostin produced both at the mRNA and protein level in MLO‐A5 cells (Kato et al., 2001). Also, sclerostin is a marker of the late osteocyte (Winkler et al., 2003) and as our results here are promoting differentiation of osteoblasts into immature osteocytes, we do not think that the 24 h time period is enough to push the cells into a more mature sclerostin‐expressing osteocyte phenotype. Osteocyte function is known to be tightly linked to the Wnt/β‐catenin signaling pathway. Since key components of this pathway, such as β‐catenin, as well as those of other crucial pathways in bone, including NF‐κB, are known to be regulated by proteasomal turnover, it is possible that this new proposed key role for the proteasome in osteocyte form and function may be more extensive (Gaur et al., 2005; Skaug et al., 2009). Indeed whilst our data here suggests E11 to be the likely candidate, it is possible that other mechanisms may be involved which again, must be considered should E11 stabilization be pursued as a therapeutic target.

One of the most potent promoters of E11 protein levels observed herein was Bortezomib. Bortezomib, a peptide boronic acid congener, directly inhibits the chymotrypsin‐like activity of the proteasome PSMB5 subunit and is used for the treatment of multiple myeloma; it is also undergoing clinical trials for the treatment of several epithelial cancers (Ria et al., 2014). Bone disease occurs in up to 80% of patients with multiple myeloma and is characterised by an imbalance in bone remodelling towards increased osteoclastic bone resorption (Qiang et al., 2012). Proteasome inhibition in patients with multiple myeloma has an anabolic effect on bone formation (Garrett et al., 2003; Zangari et al., 2005; Terpos et al., 2007), thought to be through its effects on β‐catenin and the NF‐κB pathway (Qiang et al., 2009). Bortezomib also increases osteogenic differentiation and bone formation in mesenchymal stem cells and C2C12 cells, highlighting its potential as a therapeutic agent for other diseases of bone loss (Giuliani et al., 2007; Uyama et al., 2012; Sanvoranart et al., 2014). Whether these anabolic effects of proteasome inhibitors on bone formation are through stabilization of E11 is yet to be determined. It would be of particular interest to investigate bone morphology in E11 null mice treated with proteasome inhibitors. Such mice are however embryonically lethal (Zhang et al., 2006), and as such the most revealing studies would be those conducted in an osteocyte specific conditional E11 knockout mouse.

As well as the observed increase in E11 protein expression, we also revealed that Bortezomib/ALLN/MG132 increased *E11* mRNA levels. In other studies MG132 and Bortezomib have been reported to induce mRNA increases through both transcriptional (promoter activation) and post‐transcriptional (mRNA stability) mechanisms (Butler et al., 2009; Laroia et al., 1999; Shimizu et al., 2013). Conversely, MG132 causes defective polyadenylation (Lee and Moore, 2014) and whilst it has been previously shown that in human tissues, there is a 2.7 kb and a 0.9 kb E11 mRNA species, which differ in their polyadenylation (Martin‐Villar et al., 2009), there is no suggestion that this is the case in murine tissues. It would be interesting however to examine whether the stabilization of E11 observed here is maintained upon addition of a protein synthesis inhibitor, such as cycloheximide. However, the known off target effects of such a pharmacological agent are sufficient to render the merits of such experimentation questionable. Similarly, it would be interesting to do knockdown studies of E11 in proteasome inhibitor treated cells, however our numerous efforts using several commercially available siRNA constructs have allowed us only to partially knockdown E11 and as such, the residual E11 would make it very difficult to interpret any resultant data.

Ubiquitin is conjugated to proteins that are destined for proteasome‐mediated degradation by three broadly expressed enzymes: (1) ubiquitin‐activating enzyme (E1), (2) ubiquitin‐conjugating enzyme (E2), and (3) ubiquitin ligase (E3). In bone, runt‐related transcription factor 2 (RUNX2) is known to be tightly controlled by the proteasome through the E3 ubiquitin ligases Smad ubiquitin regulatory factor ‐1 (Smurf‐1), Smurf‐2 and WW‐domain‐containing protein 1 (WWP1) (Zhao et al., 2004, 2003). Here, immunoprecipitation demonstrated that E11 is also ubiquitinated in MLO‐A5 cells. We also show the increased expression of ubiquitinated‐E11 upon treatment of cells with MG132 and Bortezomib. E11 is a hydrophobic transmembrane protein in which O‐glycosylation confers resistance to proteolytic degradation; indeed blockade of O‐glycosylation in endothelial cells markedly diminishes E11 levels and intriguingly phenocopies the global E11^−/−^ embryonic mouse (Fu et al., 2008). Indeed it is well recognised that protein ubiquitination is modulated by glycosylation (Roos‐Mattjus and Sistonen, 2004; Butkinaree et al., 2010) and as such, this raises the possibility that E11 glycosylation enhances E11 stability in pre‐osteocytes; a possibility that is currently being explored.

To further delineate the mechanisms by which stabilization of E11 promotes osteocyte differentiation, we have investigated potential downstream signaling and function, namely the RhoA pathway. RhoA is a small GTPase and a master regulator of various cellular processes such as cytokinetics, cytoskeletal regulation, and cell migration (Takai et al., 2001). In addition to osteocytes, E11 has also been detected in many other cells and it was in one of these cell types, MDCK, that E11 was first demonstrated to regulate RhoA signaling (Martin‐Villar et al., 2006). Activation of RhoA by E11 was required for the formation of cell protrusions and invasion of the cells through a matrigel substrate. To investigate whether E11 was able to activate RhoA in MLO‐A5 cells it was first necessary to examine small GTPase expression in this cell line. RhoA protein was detected at all stages of MLO‐A5 cell differentiation and its activation (GTP‐bound RhoA) was increased in MLO‐A5 cells from day 3 of culture. This pattern mimicked that of E11, osteocyte differentiation and matrix mineralization in these cells. This suggests a link between E11 expression and increased activation of RhoA in MLO‐A5 cells, similar to those previously observed in MDCK cells. It also suggests that RhoA activation may be required for osteocytogenesis, as was further indicated by the increased RhoA activity observed upon E11 stablization. The role of RhoA signaling during osteoblast differentiation is however a contentious issue, with studies describing both a positive (McBeath et al., 2004; Meyers et al., 2005; Arnsdorf et al., 2009; Khatiwala et al., 2009) and negative (Harmey et al., 2004; Kanazawa et al., 2009) effect of RhoA activation on osteoblast differentiation.

RhoA has many downstream effectors including ROCK which has been shown to be important in the cytoskeletal changes observed in podoplanin induced MDCK cell epithelial‐mesenchymal transitions (Martin‐Villar et al., 2006). Here cells were cultured with the selective ROCK inhibitor H‐1152 in the presence (or absence) of ALLN. MLO‐A5 cells cultured with ROCK inhibitor alone displayed a flattened and spread morphology with few processes. It would therefore be reasonable to expect ROCK inhibition to have an antagonistic effect on the formation of processes in response to proteasome inhibition. The opposite was true however, as MLO‐A5 cultures supplemented with ROCK inhibitor and ALLN displayed a particularly pronounced dendritic morphology similar, but quantitatively more marked, than in cells treated with ALLN alone. MLO‐A5 cells from the ROCK and ALLN supplemented cultures also projected a greater number of processes compared to cultures treated with ALLN alone and the processes showed a higher degree of connectivity, forming an in vitro interconnected network of cells. The reasons for the additive effects of both proteasome and ROCK inhibition are unknown, but a recent study has shown that ROCK II impairs dendrite formation and growth in neurons (Duffy et al., 2009). If ROCK II has similar functions in osteocytes then this suggests a possible mechanism of regulating E11‐induced process formation. This suggests that increases in RhoA signaling alone may not be responsible for the ALLN‐mediated changes in cell structure. Another cytoskeletal protein ezrin, which is a member of the ERM family of proteins and is essential for linkage of the actin cytoskeleton to the plasma membrane, is also involved in the formation of filopodia by activating RhoA and this occurs due to binding of ezrin to specific binding sites within E11 (Scholl et al., 1999; Martin‐Villar et al., 2014, 2006). This could also provide explanation as to the increased ERM observed in our cultures treated with both the ROCK inhibitor H‐1152 and proteasome inhibitors.

Together, these data suggest that proteasome‐mediated degradation of E11 acts to suppress Rho‐mediated process formation. Perhaps this provides evidence for a point of integration of E11 and Rho pathways, at which multiple signals converge to accelerate osteocytogenesis. These findings may indicate that this osteocytogenic process also initiates mechanisms that endogenously suppress excessive E11‐related process formation. Further experiments are required however, before the role of RhoA and ROCK during osteocytogenesis may be fully elucidated.

In summary, our data indicate that E11 protein is essential for osteocyte formation, and our understanding that proteasome‐mediated E11 protein degradation limits this acquisition of the osteocyte phenotype will offer new insights into the process of osteocytogenesis. Considering the use of Bortezomib in clinics, our findings in this study warrant further investigations to determine whether proteasome inhibitors could be used for other bone‐related diseases.
